# A novel use of an artificially intelligent Chatbot and a live, synchronous virtual question-and answer session for fellowship recruitment

**DOI:** 10.1186/s12909-022-03872-z

**Published:** 2023-03-11

**Authors:** Peter K. Yi, Neil D. Ray, Noa Segall

**Affiliations:** Department of Anesthesiology and Critical Care, Duke University School of Medicine, Durham, North Carolina USA

**Keywords:** Innovation and technology, Graduate medical education, Recruitment, Social media, Artificial intelligence

## Abstract

**Introduction:**

Academic departments universally communicate information about their programs using static websites. In addition to websites, some programs have even ventured out into social media (SM). These bidirectional forms of SM interaction show great promise; even hosting a live Question and Answer (Q&A) session has the potential for program branding. Artificial Intelligence (AI) usage in the form of a chatbot has expanded on websites and in SM. The potential use of chatbots, for the purposes of trainee recruitment, is novel and underutilized. With this pilot study, we aimed to answer the question; can the use of an Artificially Intelligent Chatbot and a Virtual Question-and-Answer Session aid in recruitment in a Post-COVID-19 era?

**Methods:**

We held three structured Question-and-Answer Sessions over a period of 2 weeks. This preliminary study was performed after completion of the three Q&A sessions, in March–May, 2021. All 258 applicants to the pain fellowship program were invited via email to participate in the survey after attending one of the Q&A sessions. A 16-item survey assessing participants’ perception of the chatbot was administered.

**Results:**

Forty-eight pain fellowship applicants completed the survey, for an average response rate of 18.6%. In all, 35 (73%) of survey respondents had used the website chatbot, and 84% indicated that it had found them the information they were seeking.

**Conclusion:**

We employed an artificially intelligent chatbot on the department website to engage in a bidirectional exchange with users to adapt to changes brought on by the pandemic. SM engagement via chatbot and Q&A sessions can leave a favorable impression and improve the perception of a program.

**Supplementary Information:**

The online version contains supplementary material available at 10.1186/s12909-022-03872-z.

## Background

The COVID-19 pandemic has brought sweeping changes to how we recruit residents and fellows. Due to travel restrictions, social distancing and limits on gatherings, the norm of in-person interviews has been replaced by virtual interview platforms. Multiple regulatory bodies, including the Accreditation Council for Graduate Medical Education (ACGME), have called accredited programs to “commit to online interviews and virtual visits” (https://www.aamc.org/system/files/202005/covid19_Final_Recommendations_Executive%20Summary_Final_05112020.pdf). Although web based interviews (WBIs) have substantial cost and time benefits, they do pose their own new challenges; mainly due to the lack of in-person, face-to-face interactions and the difficulty in picking up non-verbal cues on a screen [1, 2]. However, despite these short-comings, WBIs may signal the start of changes and innovation to the traditional interview format and may even become a commonly used modality post-pandemic. As Graduate Medical Education programs pivot towards WBIs, a focus on other virtual recruitment tools has also gained traction.

In addition to WBIs, recruitment tactics have also shifted to more digital media-centric content [2]. Academic departments universally communicate information about their programs and about the department in general, using static websites. Residency websites have been shown to influence applicants’ first impression of a program and are a critically important factor in decision making [3]. In addition to websites, some programs have even ventured out into social media (SM) such as Twitter and Instagram to offer more engaging and detailed content [4]. Most applications of this type of SM are unidirectional, meaning that the host specifically curates information to present. However, opportunities for bidirectional communication can potentially pave the way for additional innovative uses. Very few programs use this type of SM interaction either in an asynchronous or synchronous manner. An example of synchronous, bidirectional use of SM is Google Hangouts [5]. These bidirectional forms of SM interaction show great promise; even hosting a live Question and Answer (Q&A) session has the potential to improve the overall perception of a program. Hass, et al. recently mentioned that traditional strategies of recruitment including branding, fostering a national reputation, developing a website, and utilizing a SM presence have been emphasized in the past [2]. The COVID-19 pandemic brought upheaval to these traditional methods of recruitment and has forced us to reimagine innovative ways to recruit residents and fellows. Virtual program branding has become essential and SM use, along with Q&A sessions, could be a key component to the type of branding that is necessary for virtual residency recruitment [6].

SM appears to have an increasing influence on residency decisions [7]. With the concurrent expansion of SM, technological development in the realm of Artificial Intelligence (AI) has grown exponentially as well. AI is a field which combines computer science and robust datasets, to enable problem-solving (https://www.ibm.com/cloud/learn/what-is-artificial-intelligence). With the growth of AI, its effects have permeated into various aspects of our lives, including the medical field. AI is changing traditional methods of medical imaging analysis, health data acquisition, and even medical education. AI has practical uses for distance learning and various inquiry systems, however AI for the application of trainee recruitment has not been reported [8]. AI usage in the form of a chatbot or virtual concierge agent has expanded on websites and in SM. A chatbot is defined as “a computer program designed to simulate conversation with human users, especially over the Internet” and is also known as a smart bot or digital assistant [9]. Chatbots can mimic human conversation and are useful in applications such as information retrieval and education [10]. While there are uses of chatbot as recruiters in the business world, there are no reports of chatbot use in Graduate Medical Education [10]. While much of the technology behind these uses are continuously being developed and applied, the potential use of AI and chatbots, for the purposes of trainee recruitment, is novel and underutilized. The proposed benefits of AI include relieving and splitting work performed by humans as well as replacing and augmenting human work [11]. AI and chatbots are continuously pushing the limits of the tasks machines can take over from humans and can potential save time and human resources.

In order to adapt to changes brought on by the pandemic, we recently employed an artificially intelligent chatbot on the department website to engage users in a bidirectional exchange. The chatbot is a conversational AI agent which uses natural language to understand phrases. It was tested by a third party to create the appropriate flow of information and allow users to ask questions and search for information. For example, applicants could ask the chatbot “Show me faculty that trained at <applicants home residency> or where do fellows live?” The chatbot would then return profiles of faculty that met criteria and an interactive google map of where fellows live, respectively. In addition, we used the chatbot to schedule and coordinate live, synchronous Question and Answer sessions about our program. Instead of using separately purchased scheduling software which would still rely on human monitoring, we employed the use of a chatbot to aid in some of the work usually performed by a human coordinator. We used these new technologies to complement the traditional strategies that are used to engage potential applicants. With this preliminary study, we aimed to answer the questions; can the use of an Artificially Intelligent Chatbot and a Virtual Question-and-Answer Session aid in recruitment in a Post-COVID-19 era and how will AI change what tasks machines can perform?

## Methods

We held three structured Question-and-Answer Sessions over a period of 2 weeks. These sessions were coordinated to be live and synchronous. Invites to these sessions were sent out to all applicants to our program through the Electronic Residency Application Service (ERAS) email contact information provided. Applicants were instructed to sign up through the chatbot for one of three Q&A sessions. The chatbot registered each invitee and sent out a calendar invite with a direct web link to the Q&A session. This way each applicant that signed up would have a reminder for our Q&A session with a link on their calendars. These sessions were held 2 months prior to sending out interview invitations and participation had no bearing on the decision to offer an interview. Each 30 minute session briefly covered the mission and vision of the division (led by Division Chief), highlighted the local area and attractions (led by Program Coordinator who is local to the area), then described clinical sites and the details of the fellowship throughout the year (led by Program Director). The moderators discussed research opportunities and emphasized the multidisciplinary aspect of training. Finally, participants were given a chance to ask any program specific questions.

This pilot study was performed after completion of the three Q&A sessions, in March–May, 2021. All 258 applicants to the pain fellowship program were invited via email (by a third party) to participate in the survey. A 16-item survey assessing participants’ perception of the chatbot was administered online via Qualtrics. The items were adapted from the System Usability Scale [12] and from a survey for gauging consumers’ perceptions of chatbots (https://www.userlike.com/en/blog/consumer-chatbot-perceptions). If participants indicated that they participated in a Q&A session, they were presented with 7 additional items (see Additional file 1) that evaluated the usefulness of the sessions and their effect on participants’ attitudes toward the pain fellowship program. Questions were designed to gauge ease of use with the chatbot and evaluate a participant’s overall perception of the program. We used descriptive statistics to gauge these constructs. The study protocol was approved by the Institutional Review Board.

## Results

Forty-eight pain fellowship applicants completed the survey, for an average response rate of 18.6%. In all, 35 (73%) of survey respondents had used the Department of Anesthesiology website chatbot, and 84% indicated that it had found them the information they were seeking. Respondents often asked the chatbot multiple questions, with 31% asking at least 5 questions.

Not surprisingly, most survey respondents used the chatbot to find information about the fellowship program (69%), and many also sought to learn about the University (41%), the Anesthesiology Department (37.5%), and its residency program (28%). 37.5% of respondents used the chatbot to sign up for a Q&A session. Less often, they used the chatbot to find people in the department, to learn about the city, and where the program is located. Of the chatbot’s features, 31% of respondents found scheduling a Q&A session most appealing and an additional 31% found asking it questions to be the most appealing feature. Another 22% found all features appealing and 6% found none to be appealing.

**Fig. 1:**
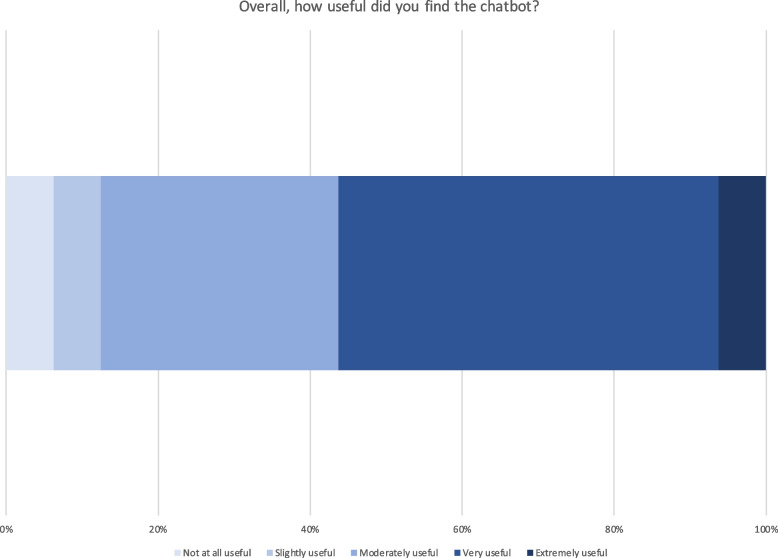
Chatbot usefulness question

**Fig. 2:**
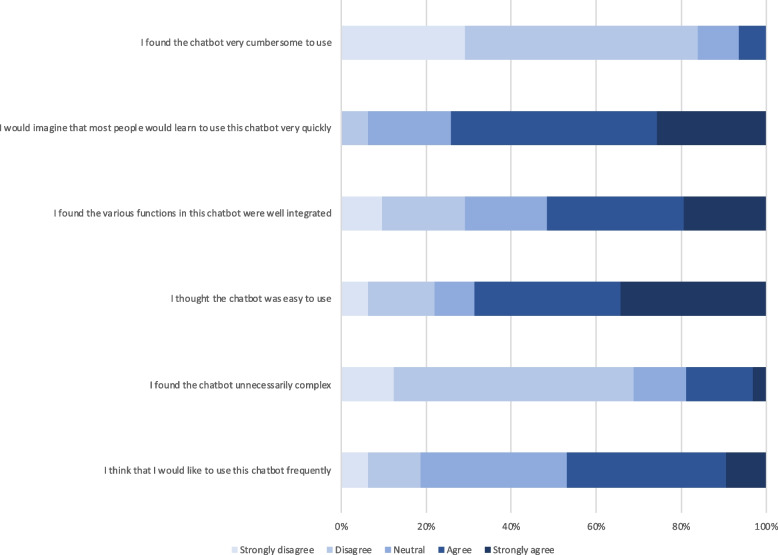
Chatbot usability questions

**Fig. 3:**
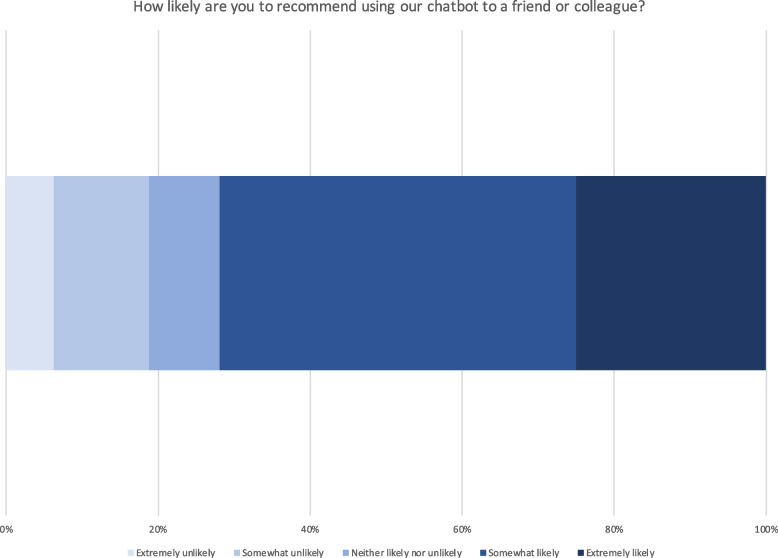
Question about recommending the chatbot to others

**Fig. 4:**
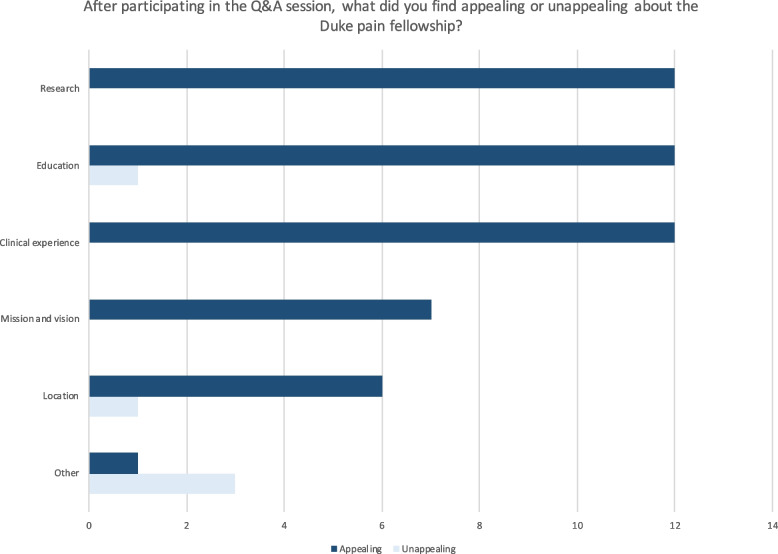
Appealing and unappealing features of the pain fellowship program

Survey questions also targeted the chatbot’s advantages and disadvantages. Most respondents perceived the chatbot to provide a quick (84%) or helpful (56%) response, to be a cool new technology (66%), and to be friendly (50%). In terms of disadvantages, 22% indicated the chatbot was unable to help them, 11% said that finding the information they needed required a prolonged effort, and the chatbot couldn’t understand an additional 11% of survey respondents. However, 59% of respondents found no disadvantages to the chatbot. Overall, respondents seemed to find conversations with the chatbot a positive experience and the chatbot to be useful and usable (Figs. 1, 2, 3 and 4).

Seventeen (39%) survey respondents participated in a Q&A session out of a total of 58 participants who logged on for a Q&A session. Of these, 93% participated to learn more about the pain fellowship program and 87% to show interest in the program. Q&A session participants indicated they learned more about the program from the Q&A session than from the Department Anesthesiology website (73%) or that both sources of information were equally useful (27%); none found the website more useful for learning about the program than the Q&A session. The perception of the pain fellowship program improved for 87% of participants after the Q&A session and made them more likely to interview for the program. Many found the research, education, and clinical experience provided to be appealing; a minority found the education and location of the program to be unappealing (Fig. 4).

## Discussion

Using AI technology, in the form of a chatbot, to provide auxiliary information appears to have advantages. Once participants landed on the website, they did use the chatbot frequently to ask specific questions. They found the chatbot to be more useful than the information found on the website and interacted with the chatbot for multiple questions. Using the chatbot to coordinate Q&A sessions was well accepted by applicants. Finally, hosting a live, synchronous Q&A session scheduled through the chatbot, had an overwhelmingly positive effect on the perception of the program. Participants found many aspects of the program appealing after the Q&A session and were more likely to interview if given the opportunity.

We know that interview days heavily influence match list rankings giving applicants an overall impression of the atmosphere of the program [13]. The loss of this opportunity prompts a need to look at alternatives to the in-person interview to allow applicants to gauge the suitability of the program. Using technology and taking advantage of SM may be a way to fill this void. We know that use of SM is one method for a program to increase applications and interviews as well as increase a program’s position on a rank list [14]. However, few programs take full advantage of SM for these purposes or even for the intent to give more insight about a program after pivoting to virtual interviews [3]. Taking additional steps to incorporate AI and a chatbot may be another way for programs to maximally utilize SM and offer a way to engage.

While Machine Learning and AI have been incorporated into certain aspects of medical education such as procedural assessment tools for cases involving image analysis [15], to our knowledge, there have been no other studies examining the use of AI integrated into SM for the use of applicant recruitment. While this pilot study is interesting and useful, more studies should look at this emerging technology for the purposes of potential recruitment. Future study efforts could focus on more specific outcomes related to matriculation and also explore how much time and resources an AI Chatbot could ultimately save. Limitations of this study include a low response rate, making it difficult to generalize participant responses to all fellowship applicants. Demographic and social media use information on survey participants could have been analyzed to further tease out trends. Although we explicitly stated the survey had no bearing on the application process, participants may have believed that completing the survey would increase their chance of acceptance.

## Conclusions

With an increase in SM use, especially due to the pandemic, a program’s website and SM platforms can have a significant impact on recruitment and application decisions [16]. Incorporating AI chatbots may provide additional information to applicants and reduce the need for human intervention for certain tasks. The development and implementation of chatbots are relatively easy with many industry sponsored platforms available to seamlessly incorporate into SM. Chatbots may therefore be a way to engage potential applicants [17]. Q&A sessions are also a relatively simple way to offer bidirectional interaction with applicants. SM engagement can leave a favorable impression and improve the perception of a program; both chatbots and virtual live Q&A sessions may be creative ways to optimally use this existing technology.

## Supplementary Information

**Additional file 1:** Survey Questions.

## Data Availability

Please contact Peter Yi at peter.yi@duke.edu for data.

## Abbreviations

ACGME: Accreditation Council for Graduate Medical Education
AI: Artificial Intelligence
ERAS: Electronic Residency Application Service
Q&A: Question and Answer
SM: Social media
WBIs: Web based interviews
