# Supplementary material for: A novel use of an artificially intelligent Chatbot and a live, synchronous virtual question-and answer session for fellowship recruitment
Source: BMC Med Educ. 2023 Mar 11;23:152. doi: 10.1186/s12909-022-03872-z (PMC10006550; doi:10.1186/s12909-022-03872-z)
Supplement: Supplementary file 1 — Additional file 1: Survey Questions. [file 12909_2022_3872_MOESM1_ESM.docx]

Survey Questions

Q1 Did you chat with Blue, the Duke Anesthesiology chatbot?

Q2 What were some of the chatbot's advantages?

Q3 What were some of the chatbot's disadvantages?

Q4 For what purpose(s) did you use the chatbot?

Q5 What feature of the chatbot did you find most appealing?

Q6 Did the chatbot provide you with the information you were looking for?

Q7 In all, how many questions did you ask the chatbot?

Q8 Overall, how useful did you find the chatbot?

Q9 For each of the following statements, please mark one choice that best describes your reactions to the chatbot.

|  | Strongly disagree (1) | (2) | (3) | (4) | Strongly agree (5) |
| --- | --- | --- | --- | --- | --- |
| I think that I would like to use this chatbot frequently (1) |  |  |  |  |  |
| found the chatbot unnecessarily complex (2) |  |  |  |  |  |
| I thought the chatbot was easy to use (3) |  |  |  |  |  |
| I found the various functions in this chatbot were well integrated (4) |  |  |  |  |  |
| I would imagine that most people would learn to use this chatbot very quickly (5) |  |  |  |  |  |
| I found the chatbot very cumbersome to use (6) |  |  |  |  |  |

Q10 How likely are you to recommend using our chatbot to a friend or colleague?

Q11 Please share any additional feedback that could help us improve your experience of our website.

Q12 How often did you use the chatbot for specific questions about the Duke Fellowship or Residency?

Q13 Was signing up for a Q&A session or for a virtual chat easier or harder with the chatbot than using other methods?

Q14 Did the chatbot help you learn more about a fellowship or residency than you found on the website?

Q15 What was your reason for not using the chatbot?

Q16 Did you participate in a Q&A session?

Q17 Why did you participate in the Q&A session?

Q18 How helpful was the Q&A session compared to information on the Duke Anesthesiology website for learning about the Duke pain fellowship?

Q19 How did your perception of the Duke pain fellowship change after the Q&A session?

Q20 Did the Q&A session make you more or less likely to interview for the Duke pain fellowship program?

Q21 After participating in the Q&A session, what did you find appealing about the Duke pain fellowship?

Q22 After participating in the Q&A session, what did you find unappealing about the Duke pain fellowship?

Q23 What made clinical experience in the Duke pain fellowship appealing or unappealing to you?
